# Antibiotic Prevention for Maternal Group B Streptococcal Colonization on Neonatal GBS-Related Adverse Outcomes: A Meta-Analysis

**DOI:** 10.3389/fmicb.2017.00374

**Published:** 2017-03-17

**Authors:** Shunming Li, Jingya Huang, Zhiyao Chen, Dan Guo, Zhenjiang Yao, Xiaohua Ye

**Affiliations:** School of Public Health, Guangdong Pharmaceutical UniversityGuangzhou, China

**Keywords:** group B *Streptococcus*, infection, intrapartum antibiotic prophylaxis, clinical adverse outcomes, meta-analysis

## Abstract

Maternal colonization with group B *Streptococcus* (GBS) during pregnancy increases the risk of neonatal infection by vertical transmission. However, it remains unclear whether treating all colonized women during labor exposes a large number of their neonates to possible adverse effects without benefit. We performed a meta-analysis to assess the effect of intrapartum antibiotic prophylaxis on neonatal adverse outcomes. We identified studies by searching several English and Chinese electronic databases and reviewing relevant articles. Data were pooled using fixed-effects or random-effects meta-analysis, and for each outcome both risk ratio (RR) and 95% confidence intervals (95% CIs) were calculated. Fourteen studies (2,051 pregnant women and 2,063 neonates) were included, comprising 13 randomized clinical trials and 1 cohort study. Antibiotic prophylaxis is associated with a significant reduced risk of all cause infections (RR = 0.28, 95% CI = 0.18–0.42), GBS infection (RR = 0.24, 95% CI = 0.13–0.44), early-onset GBS infection (RR = 0.24, 95% CI = 0.13–0.45), non-GBS infections (RR = 0.34, 95% CI = 0.20–0.59), and GBS colonization (RR = 0.10, 95% CI = 0.06–0.16). But no significant reduction was observed in late-onset GBS infection, mortality from early-onset GBS infection or from non-GBS infections. Notably, no significant differences were found between ampicillin and penicillin prevention for neonatal adverse outcomes. Our findings suggest that antibiotic prophylaxis is effective in reducing neonatal GBS colonization and infection.

## Introduction

Group B *Streptococcus* (GBS) is a significant cause of neonatal sepsis and meningitis and of severe infections in pregnant women (Murayama et al., 2009). GBS is also a commensal that colonizes the gastrointestinal and genitourinary tracts, resulting significant maternal and perinatal morbidity, and can be transmitted from a GBS-colonized mother to her newborn via the ascending route during labor and delivery, causing neonatal severe invasive diseases such as an early-onset GBS (EOGBS) disease occurring within the first week and a late-onset GBS (LOGBS) disease occurring between 1 week and 3 months of life (Borchardt et al., 2006; Huber et al., 2011; Stoll et al., 2011; Okike et al., 2014). American Centers for Disease Control (CDC) had published three guidelines in the past 20 years (1996, 2002, and 2010) that recommend the use of a risk-based or screening-based approach to identify candidates for intrapartum antibiotic prophylaxis (IAP; Centers for Disease Control and Prevention (CDC), 1996; Schrag et al., 2002; Verani et al., 2010).

It is important to know if IAP does more good than harm in trying to reduce mortality and morbidity from neonatal GBS infection. However, the potential efficacy of IAP is not clear. A few studies reported a significant inverse association between IAP and neonatal GBS-related adverse outcomes (including GBS infection and early-onset GBS infection), while other studies found no significant association between the two (Matorras et al., 1991; Gervasio et al., 2001; EI Helali et al., 2009; Li and Meng, 2010). In addition, there were a few quantitative reviews evaluated the efficacy of IAP for women with specific diseases [preterm premature rupture of membranes (PROM) or chorioamnionitis; (Egarter et al., 1996; Benitz et al., 1999)], but the potential preventive effect for healthy women is still unclear. Therefore, we systematically identified RCT and cohort studies on the issue published up to April 2016, and carried out a meta-analysis to explore the potential effect of intrapartum antibiotics for maternal GBS colonization on neonatal adverse outcomes (including GBS colonization, infection, and mortality).

## Methods

### Search strategy

The meta-analysis was conducted following the PRISMA guidelines (Moher et al., 2010). Studies were identified through a systematic search of MEDLIINE, PUBMED, WANGFANG, and CNKI, from January 1979 to April 2016, using the following searching terms: (“group B *streptococcus*” or “streptococcus agalactiae”) and (“antibiotic” or “anti-bacterial agents” or “intrapartum antibiotic prophylaxis”) and (“perinatal period” or “pregnant women”). In addition, reference lists of all retrieved articles and previous systematic reviews were checked for further eligible publications. We restricted our search to studies performed in human studies and published in English or Chinese.

### Inclusion and exclusion criteria

Two investigators (SM Li and JY Huang) independently reviewed and assessed the eligibility of identified studies using the following inclusion and exclusion criteria. The inclusion criteria required studies to: (1) include original data from RCT or cohort studies; (2) be based on pregnant women known to be colonized with GBS in the vaginal/ intestinal tract and/or the urinary tract at any time during the pregnancy; (3) provide information on intrapartum use of antibiotics; (4) assess the impact of antibiotics on clinical outcomes of neonates; and (5) apply treatment or placebo explicitly and equally to both treatment and control participants. Studies were excluded if they: (1) were case reports, letters, abstracts, reviews, or meta-analyses; (2) did not mention how many participants were allocated to treatment or control group; and (3) administered intrapartum treatment to both treatment group and control group. When multiple articles reported the same study population, only the most recent and informative study that met inclusion criteria was included.

### Outcomes of interest

The outcomes of interest included: (1) neonatal mortality (including all cause mortality, mortality from EOGBS infection or infections caused by bacteria other than GBS); (2) neonatal infection [including all cause infections, GBS infection, EOGBS infection (symptoms and signs of sepsis or pneumonia in a neonate born to a GBS positive mother, and positive GBS bacterial cultures which from normally sterile body fluids obtained from the neonate), LOGBS infection (infection due to GBS in an infant at least seven days old), and infections caused by bacteria other than GBS]; and (3) neonatal GBS colonization (swabs sampled in external ear, nose, throat, umbilicus, and rectum within 24 h of birth were cultured GBS positive).

### Data extraction

Data extracted from each study included the name of the first author, publication year, country, study design, population source, number of participants, antibiotic use, and adverse outcomes of neonates. Detailed information is shown in Table 1 and Table S1. Two investigators (SM Li and JY Huang) independently reviewed and cross-checked the data, and disagreements were resolved by consensus.

**Table 1:** **Main characteristics of studies in the meta-analysis**.

**Author, year**	**Country**	**Study design**	**Quality index^a^**	**Antibiotic use**	**Neonate**	
					***n*_1_/*n***	**Adverse outcomes^b^**
Matorras et al., 1991	Spain	RCT	2	AMP	60/121	NGBSC, EOGBSI, LOGBSI, NI
Gervasio et al., 2001	USA	Cohort	7	PEN	345/451	EOGBSI,MGBSI,MI
EI Helali et al., 2009	France	RCT	1	PEN	63/126	NGBSC,EOGBSI
Li and Meng, 2010	China	RCT	1	PEN	136/227	NGBSC,EOGBSI
Yow et al., 1979	USA	RCT	1	AMP	34/58	NGBSC
Easmon et al., 1983	British	RCT	1	PEN / ERY	38/87	NGBSC
Lim et al., 1986	USA	RCT	2	AMP	80/173	NGBSC,EOGBSI
Boyer and Gotoff, 1986	USA	RCT	3	AMP	85/164	NGBSC,EOGBSI,LOGBSI, MGBSI,MI
Tuppurainen and Hallman, 1989	Finland	RCT	2	PEN	88/199	EOGBSI
Shen et al., 2012	China	RCT	1	PEN	91/117	NI
Ma et al., 2014	China	RCT	1	PEN	68/98	NI
Bai, 2014	China	RCT	2	PEN	33/65	NI
Zhang et al., 2015	China	RCT	1	PEN	92/113	NI
Yang, 2015	China	RCT	3	PEN	32/64	NI

n, number of participants; n_1_, number of participants in the treatment group; RCT: randomized controlled trial; AMP, ampicillin; PEN, penicillin; ERY, erythromycin;

a *The Newcastle-Ottawa scale was used in cohort studies, and the Jadad scale was used in RCTs*.

b *NGBSC, neonatal group B streptococcal colonization; EOGBSI, early-onset group B streptococcal infection; LOGBSI, late-onset group B streptococcal infection; MGBSI, mortality from early-onset group B streptococcal infection; NI, neonatal infections; MI, neonatal mortality from infections caused by bacteria other than GBS*.

### Quality assessment

For each study retained for the meta-analysis, we assessed methodological quality based on following criteria: (1) Randomized controlled trials were assessed using the Jadad scale; and (2) non-RCTs were assessed using the Newcastle-Ottawa quality assessment scale (NOS; Jadad et al., 1996; Wells et al., 2011). The Jadad scale awards 1–5 points and RCTs with ≥3 points are considered high-quality studies. The NOS is categorized into three dimensions (including the selection populations, comparability of groups, and outcome/exposure of interest), and non-RCTs with ≥5 points indicate high-quality studies.

### Statistical analysis

All statistical analyses were performed by using Stata statistical software version 14.0 (Stata Corporation LP, College Station, Texas, USA). All summary estimates of the relative ratio (RR) were pooled by either fixed-effects model (*P* for heterogeneity > 0.1) or random-effects model (*P* for heterogeneity ≤ 0.1; Mantel and Haenszel, 1959; DerSimonian and Laird, 1986). Heterogeneity among studies was tested by the chi-squared test with the Cochrane *Q* statistic (significant at *P* ≤ 0.1) and quantified by *I*^2^ statistic (Higgins and Thompson, 2002; Higgins et al., 2003). The subgroup analysis was performed according to maternal conditions (without infectious symptoms vs. with probable infectious symptoms), study quality (high-quality vs. low-quality), gestational ages (35–37 vs. 17–43 weeks) and antibiotic prevention (ampicillin vs. penicillin), using χ^2^-tests (significant at *P* ≤ 0.05).

## Results

### Characteristics of eligible studies

**Figure 1:**
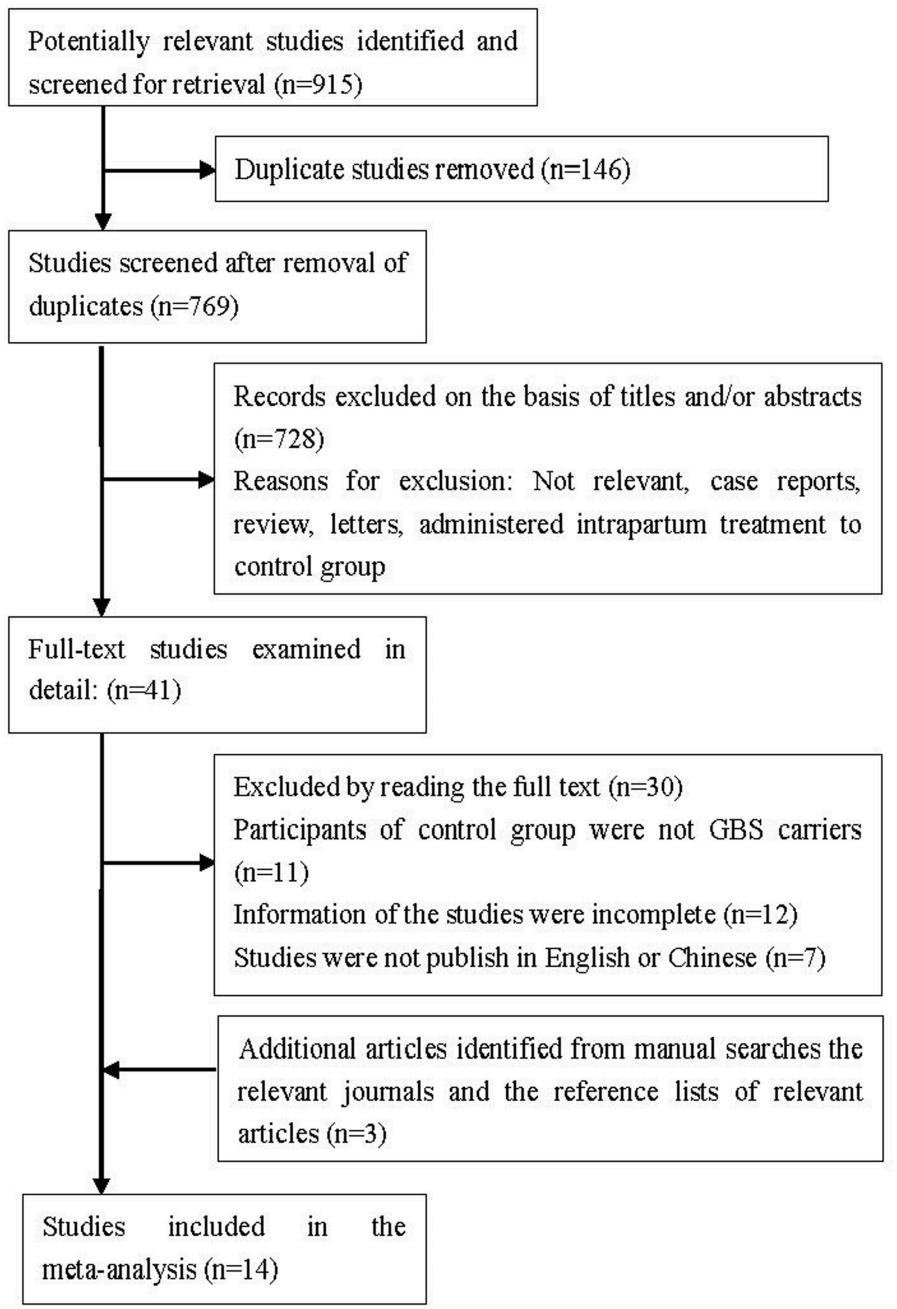
**Flowchart of literature search for meta-analysis**.

The flowchart of literature search for meta-analysis was shown in Figure 1. By reading the abstracts and using our criteria, 41 studies were selected for detailed evaluation; 30 studies were excluded for the reasons noted in Figure 1. Three studies were included through manual searches of the relevant systematic reviews and reference list (Egarter et al., 1996; Benitz et al., 1999; Ohlsson and Shah, 2014). Finally, 14 studies were included in the final analysis (Yow et al., 1979; Easmon et al., 1983; Boyer and Gotoff, 1986; Lim et al., 1986; Tuppurainen and Hallman, 1989; Matorras et al., 1991; Gervasio et al., 2001; EI Helali et al., 2009; Li and Meng, 2010; Shen et al., 2012; Bai, 2014; Ma et al., 2014; Yang, 2015; Zhang et al., 2015). The main characteristics of eligible studies are given in Table 1 and the main characteristics of participants are given in Table S1. There were 13 RCT studies and one cohort study with a total of 2051 pregnant women and 2063 neonates. Six of these studies were conducted in China, four were in USA and the rest were in Europe.

### Study quality

Thirteen manuscripts were RCTs. The maximum score in the Jadad scale was three because it is difficult to have a double-blind study in this field. Two studies received Jadad scores of 3, four studies received 2, and seven studies received 1. The only non-RCT study received NOS score of 7 (Table 1).

### Antibiotic prevention for neonatal mortality

**Figure 2:**
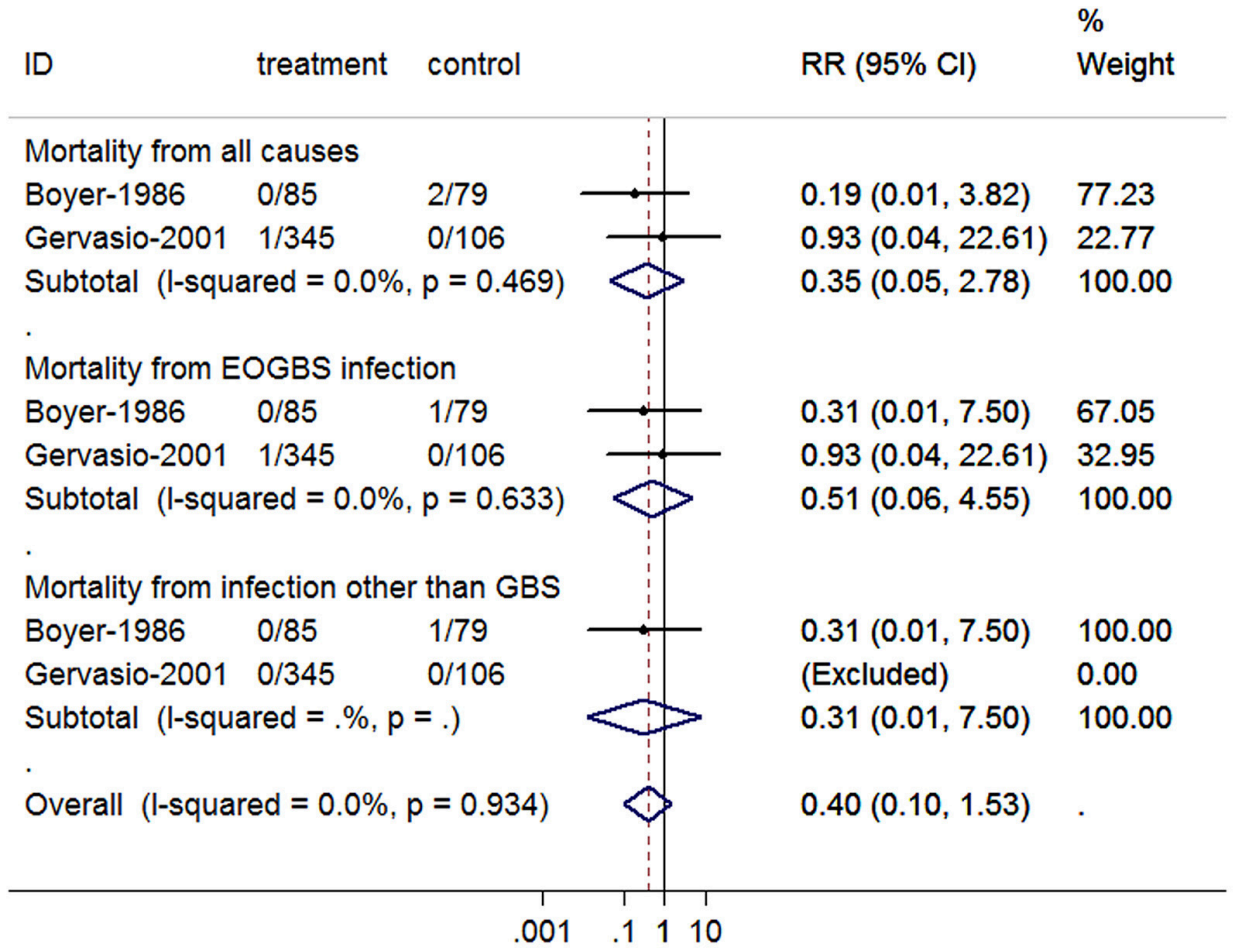
**Summary relative risk of neonatal mortality for antibiotic prophylaxis**. The combined relative risk was achieved using fixed-effects model. (GBS, group B *Streptococcus*; EOGBS, early-onset group B streptococcal).

The overall RRs of neonatal mortality for IAP were presented in Figure 2. No statistically significant reduction was observed in the incidence of all cause mortality (RR = 0.35, 95% CI = 0.05–2.78), mortality from EOGBS infection (RR = 0.51, 95% CI = 0.06–4.55) or from non-GBS infections (RR = 0.31, 95% CI = 0.01–7.50). There was no evidence of heterogeneity among studies on all cause mortality (*P* = 0.469, *I*^2^ = 0%) and mortality from EOGBS infection (*P* = 0.633, *I*^2^ = 0%).

### Antibiotic prevention for neonatal infection

**Figure 3:**
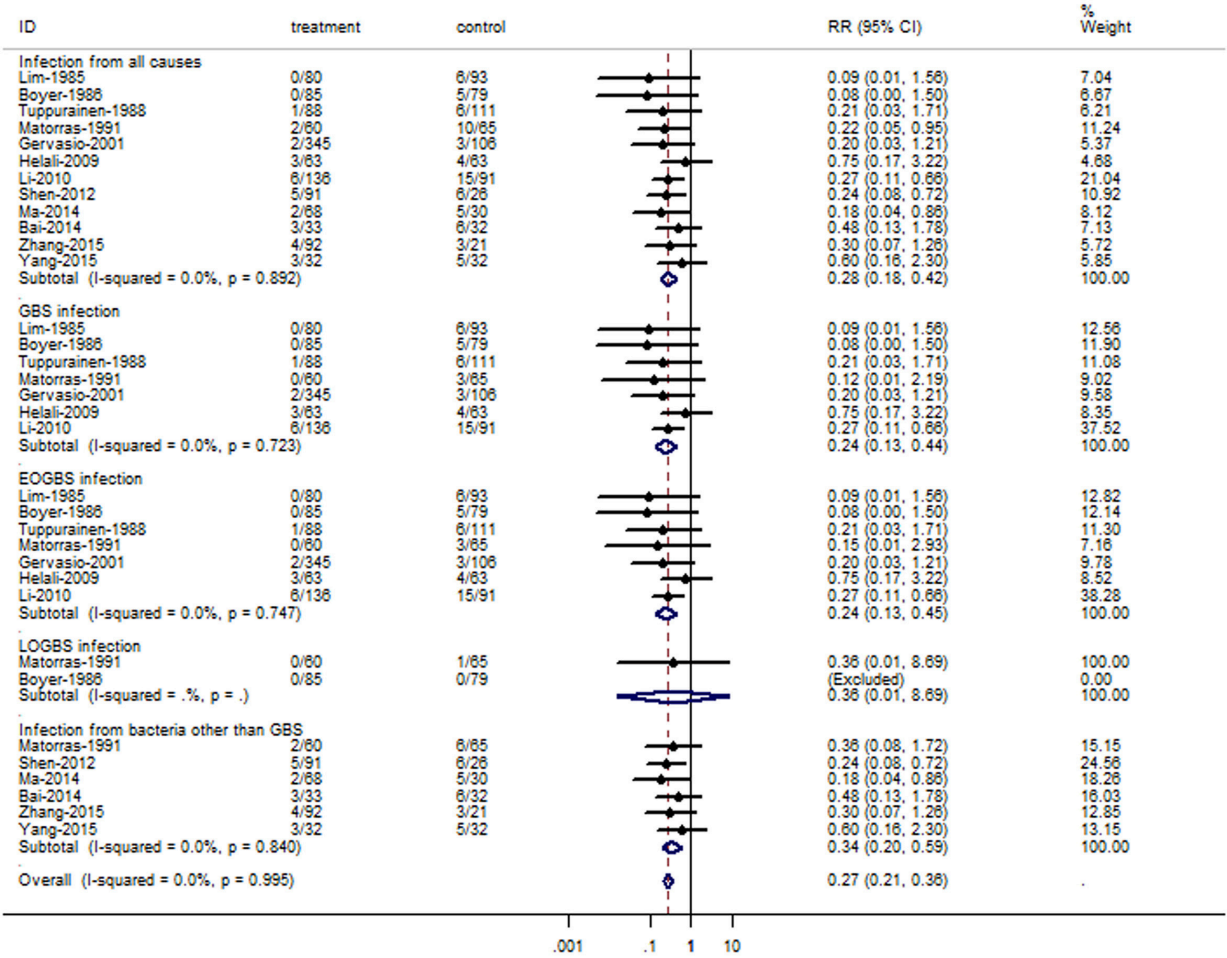
**Summary relative risk of neonatal infections cause by GBS or bacteria other than GBS for antibiotic prophylaxis**. The combined relative risk was achieved using fixed-effects model. (GBS, group B *Streptococcus*; EOGBS, early-onset group B streptococcal; LOGBS, late-onset group B streptococcal).

The overall RRs of neonatal infection for IAP were presented in Figure 3. Overall, there was a significant reduction in the incidence of all cause infections (RR = 0.28, 95% CI = 0.18–0.42), GBS infection (RR = 0.24, 95% CI = 0.13–0.44), EOGBS infection (RR = 0.24, 95% CI = 0.13–0.45), and non-GBS infections (RR = 0.34, 95% CI = 0.20–0.59) for antibiotic prophylaxis. However, no statistically significant reduction was observed in LOGBS infection (RR = 0.36, 95% CI = 0.01–8.69). There was no evidence of heterogeneity among studies on all cause infections (*P* = 0.892, *I*^2^ = 0%), GBS infection (*P* = 0.723, *I*^2^ = 0%), EOGBS infection (*P* = 0.747, *I*^2^ = 0%), and non-GBS infections (*P* = 0.840, *I*^2^ = 0%).

### Antibiotic prevention for neonatal GBS colonization

**Figure 4:**
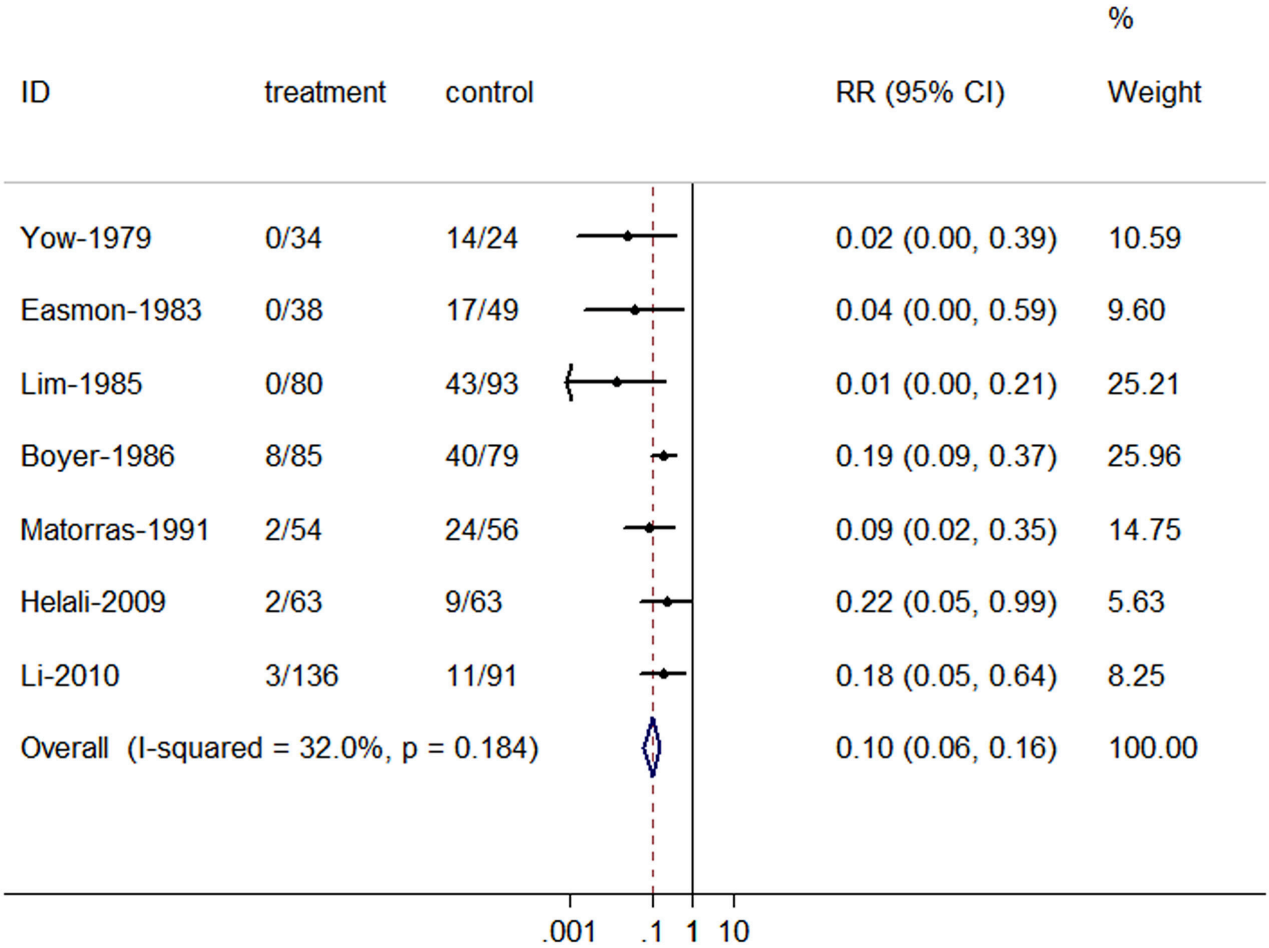
**Summary relative risk of neonatal group B streptococcal colonization for antibiotic prophylaxis**. The combined relative risk was achieved using fixed-effects model.

The overall RRs of neonatal GBS colonization for IAP were presented in Figure 4. There was a significant reduction in the incidence of neonatal GBS colonization for antibiotic prophylaxis (RR = 0.10, 95% CI = 0.06–0.16), with no evidence of heterogeneity among studies (*P* = 0.184, *I*^2^ = 32.0%).

### Comparison of efficacy of ampicillin and penicillin

In subgroup analysis by antibiotic prevention (ampicillin vs. penicillin) for neonatal adverse outcomes (Table 2), we found a similar efficacy in prevention of all cause mortality (RR = 0.19, 95% CI = 0.01–3.82, for ampicillin; RR = 0.93, 95% CI = 0.04–22.61, for penicillin; *P* for difference = 0.474), all cause infections (RR = 0.15, 95% CI = 0.04–0.48, for ampicillin; RR = 0.32, 95% CI = 0.21–0.50, for penicillin; *P* for difference = 0.272), and GBS colonization (RR = 0.08, 95% CI = 0.02–0.27, for ampicillin; RR = 0.13, 95% CI = 95% CI = 0.05–0.33, for penicillin; *P* for difference = 0.670) between two groups. When differentiating adverse outcomes in GBS-related and other bacteria related, there was still evidence of similar risks of mortality from EOGBS infection (*P* for difference = 0.634), GBS infection (*P* for difference = 0.199), EOGBS infection (*P* for difference = 0.233), and non-GBS infections (*P* for difference = 0.916) between two groups. The number of studies on other outcomes was too small to perform subgroup analysis on.

**Table 2:** **Effect comparison of subgroups of antibiotic prophylaxis**.

**Neonatal outcomes**	**Subgroups of antibiotics**	**No. of studies**	**RR(95%CI)**	**Heterogeneity *I^2^* (*P*-value)**	***P*-value for difference^a^**
Mortality from all causes	Ampicillin	1	0.19 (0.01, 3.82)	–	0.474
	Penicillin	1	0.93 (0.04,22.61)	–	
Mortality from EOGBSI	Ampicillin	1	0.31 (0.01, 7.50)	–	0.634
	Penicillin	1	0.93 (0.04,22.61)	–	
Infection from all causes	Ampicillin	3	0.15 (0.04, 0.48)	0.0% (0.768)	0.272
	Penicillin	9	0.32 (0.21, 0.50)	0.0% (0.865)	
GBS infection	Ampicillin	3	0.10 (0.02, 0.51)	0.0% (0.984)	0.199
	Penicillin	4	0.31 (0.16, 0.60)	0.0% (0.603)	
EOGBSI	Ampicillin	3	0.10 (0.02, 0.54)	0.0% (0.951)	0.233
	Penicillin	4	0.31 (0.16, 0.60)	0.0% (0.603)	
Infection from other bacteria other than GBS	Ampicillin	1	0.36 (0.08, 1.72)	–	0.916
	Penicillin	5	0.34 (0.19, 0.60)	0.0% (0.726)	
GBS colonization	Ampicillin	4	0.08 (0.02, 0.27)	57.7% (0.069)	0.670
	Penicillin	3	0.13 (0.05, 0.33)	0.0% (0.464)	

*GBS, Group B Streptococcus; EOGBSI, early-onset group B Streptococcal infection; RR, risk ratio; 95%CI, 95% confidence interval*.

a *Chi-squared test was used to test the difference of subgroups*.

### Impact of study quality, maternal condition, and gestational ages

In subgroup analysis by study quality (Table 3), we found a similar efficacy in prevention of all cause infections (*P* for difference = 0.789), GBS infection (*P* for difference = 0.474), EOGBS infection (*P* for difference = 0.464), non-GBS infections (*P* for difference = 0.349), and GBS colonization (*P* for difference = 0.258) between two groups. The number of studies on other outcomes was too small to perform subgroup analysis on. In subgroup analysis by maternal conditions (Table 4), we found a similar efficacy in prevention of all cause mortality (*P* for difference = 0.474), mortality from EOGBS infection (*P* for difference = 0.634), all cause infections (*P* for difference = 0.703), GBS infection (*P* for difference = 0.566), EOGBS infection (*P* for difference = 0.624), non-GBS infections (*P* for difference = 0.488), and GBS colonization (*P* for difference = 0.465) between two groups. In subgroup analysis by gestational ages (Table 5), we found a similar efficacy in prevention of all cause infections (*P* for difference = 0.179), GBS infection (*P* for difference = 0.351), EOGBS infection (*P* for difference = 0.390), non-GBS infections (*P* for difference = 0.279), and GBS colonization (*P* for difference = 0.502) between two groups.

**Table 3:** **Effect comparison of subgroups of study quality**.

**Neonatal outcomes**	**Subgroups of study quality**	**No. of studies**	**RR(95%CI)**	**Heterogeneity *I^2^* (*P*-value)**	***P*-value for difference^a^**
Infection from all causes	High-quality	3	0.29 (0.11, 0.76)	0.0% (0.372)	0.789
	Low-quality	9	0.28 (0.18, 0.44)	0.0% (0.884)	
GBS infection	High-quality	2	0.14 (0.03, 0.67)	0.0% (0.584)	0.474
	Low-quality	5	0.27 (0.14, 0.52)	0.0% (0.584)	
EOGBSI	High-quality	2	0.14 (0.03, 0.67)	0.0% (0.584)	0.464
	Low-quality	5	0.27 (0.14, 0.53)	0.0% (0.619)	
Infection from bacteria other than GBS	High-quality	1	0.60 (0.16, 2.30)	–	0.349
	Low-quality	5	0.30 (0.16, 0.55)	0.0% (0.881)	
GBS colonization	High-quality	1	0.19 (0.09, 0.37)	–	0.258
	Low-quality	6	0.07 (0.03, 0.14)	29.0% (0.218)	

*GBS, Group B Streptococcus; EOGBSI, early-onset group B Streptococcal infection; RR, risk ratio; 95%CI, 95% confidence interval*.

a *Chi-squared test was used to test the difference of subgroups*.

**Table 4:** **Effect comparison of subgroups of maternal conditions**.

**Neonatal outcomes**	**Subgroups of maternal condition**	**No. of studies**	**RR(95%CI)**	**Heterogeneity *I^2^* (*P*-value)**	***P*-value for difference^a^**
Mortality from all causes	Without infectious symptoms	1	0.19 (0.01, 3.82)	–	0.474
	With probable infectious symptoms	1	0.93 (0.04, 22.61)	–	
Mortality from EOGBSI	Without infectious symptoms	1	0.31 (0.01, 7.50)	–	0.634
	With probable infectious symptoms	1	0.93 (0.04, 22.61)	–	
Infection from all causes	Without infectious symptoms	6	0.25 (0.14, 0.45)	0.0% (0.658)	0.703
	With probable infectious symptoms	6	0.31 (0.17, 0.57)	0.0% (0.807)	
GBS infection	Without infectious symptoms	5	0.26 (0.13, 0.50)	0.0% (0.519)	0.566
	With probable infectious symptoms	2	0.16 (0.03, 0.80)	0.0% (0.746)	
EOGBSI	Without infectious symptoms	5	0.26 (0.13, 0.50)	0.0% (0.519)	0.624
	With probable infectious symptoms	2	0.18 (0.04, 0.89)	0.0% (0.868)	
Infection from bacteria other than GBS	Without infectious symptoms	1	0.24 (0.08, 0.72)	–	0.488
	With probable infectious symptoms	5	0.37 (0.20, 0.70)	0.0% (0.813)	
GBS colonization	Without infectious symptoms	6	0.10 (0.06, 0.17)	41.6% (0.128)	0.465
	With probable infectious symptoms	1	0.09 (0.02, 0.35)	–	

*GBS, Group B Streptococcus; EOGBSI, early-onset group B Streptococcal infection; RR, risk ratio; 95%CI, 95% confidence interval*.

a *Chi-squared test was used to test the difference of subgroups*.

**Table 5:** **Effect comparison of subgroups of gestational ages**.

**Neonatal outcomes**	**Subgroups of gestational age**	**No. of studies**	**RR(95%CI)**	**Heterogeneity *I^2^* (*P*-value)**	***P*-value for difference^a^**
Infection from all causes	35–37 weeks	7	0.34 (0.20, 0.57)	0.0% (0.755)	0.179
	17–43 weeks	5	0.19 (0.09, 0.39)	0.0% (0.974)	
GBS infection	35–37 weeks	4	0.28 (0.14, 0.57)	0.0% (0.488)	0.351
	17–43 weeks	3	0.13 (0.03, 0.54)	0.0% (0.849)	
EOGBSI	35–37 weeks	4	0.28 (0.14, 0.57)	0.0% (0.488)	0.390
	17–43 weeks	3	0.14 (0.04, 0.57)	0.0% (0.865)	
Infection from bacteria other than GBS	35–37 weeks	3	0.47 (0.21, 1.01)	0.0% (0.785)	0.279
	17–43 weeks	3	0.25 (0.11, 0.55)	0.0% (0.816)	
GBS colonization	35–37 weeks	5	0.06 (0.03, 0.14)	0.0% (0.117)	0.502
	17–43 weeks	2	0.15 (0.08, 0.28)	0.0% (0.325)	

*GBS, Group B Streptococcus; EOGBSI, early-onset group B Streptococcal infection; RR, risk ratio; 95%CI, 95% confidence interval*.

a *Chi-squared test was used to test the difference of subgroups*.

## Discussion

We contributed additionally to the literature by including more new trials to explore the potential role of antibiotic prophylaxis and differentiating neonatal adverse outcomes in GBS and non-GBS related diseases to make neonatal adverse outcomes clearer. This updated meta-analysis confirms that antibiotic prophylaxis appears to significantly reduce the risk of neonatal adverse outcomes, including all cause infections, GBS infection, EOGBS infection, non-GBS infections, and GBS colonization.

There are two types of interventions that may reduce the risk of neonatal GBS infection by vertical transmission, including for high-risk (PROM, chorioamnionitis and so on) and moderate-risk (vaginal GBS colonization, maternal fever, prematurity, and low birth weight) mothers. Although evidence from two previous meta-analyses indicated that antibiotic therapy significantly reduced the risk of sepsis in infants born to women with PROM and chorioamnionitis (Egarter et al., 1996; Benitz et al., 1999), but the potential effect for moderate-risk women is still unclear. A few available studies have explored the potential association between intrapartum antibiotics for GBS-colonized women and neonatal GBS-related infections, but current evidence is inconsistent (Boyer and Gotoff, 1986; Tuppurainen and Hallman, 1989; Shen et al., 2012; Ma et al., 2014). The only meta-analysis (four trials) on intrapartum antibiotics for maternal GBS colonization published so far indicated that incidence of EOGBS infection was reduced with intrapartum antibiotics compared to no treatment (Ohlsson and Shah, 2014). Our meta-analysis based on 14 studies provides more reliable evidence that prophylactic antibiotics for GBS-colonized women significantly reduce the risk of all cause infections, GBS infection, EOGBS infection, and non-GBS infections, indicating that antibiotic prophylaxis may reduce the risk of bacteria vertical transmission (including GBS isolates). Notably, only two RCTs conducted more than 20 years ago and enrolling a total of 289 women have been published, and as a result we found no significant reduction in LOGBS infection in our meta-analysis study. These findings highlight the need for further well designed and conducted RCTs to better understand the potential effect of antibiotic prophylaxis on LOGBS infection.

The potential effect of antibiotic prophylaxis for GBS colonized women on neonatal mortality remains unclear. It is remarkable that previous evidence on the topic has been so poorly studied, and only two studies enrolling a total of 615 women have been published (Boyer and Gotoff, 1986; Gervasio et al., 2001). Our meta-analysis on these two studies revealed that the use of intrapartum antibiotics did not significantly reduce the incidence of mortality from EOGBS infection and mortality from all causes. It is possible that the sample size was insufficient for effective assessment of intrapartum antibiotics on such low neonatal mortality (0.23% in treatment group, 1.08% in control group, for all cause mortality; 0.23% in treatment group, 0.54% in control group, for mortality from EOGBS infection; Gervasio et al., 2001; Boyer and Gotoff, 1986). With a type I error of 0.05 and power of 80% and the above incidences, 1,640 women per group and 6,894 women per group were required to detect a significant difference in mortality from all causes and mortality from EOGBS infection, respectively.

Neonatal GBS colonization is an important risk factor for the morbidity and mortality of early-onset GBS disease. Prevention of neonatal GBS colonization has been examined in several previous studies, with reduced risk ranging from 0.01 to 0.22 (Yow et al., 1979; Easmon et al., 1983; Boyer and Gotoff, 1986; Lim et al., 1986; Matorras et al., 1991; EI Helali et al., 2009; Li and Meng, 2010). Our study additionally contributes to the literature by combining these inconsistent findings and indicated that intrapartum antibiotics can prevent GBS vertical transmission from colonized mothers to their infants (RR = 0.10 for neonatal GBS colonization). Note that there are at least two mechanisms by which neonatal colonization is prevented. First, numbers of organisms in the mother's vagina and rectum are reduced temporarily so that the infant is delivered though a field that is less contaminated by GBS. Second, the level of antibiotic (ampicillin or penicillin) in the amniotic fluid remains high, so the baby is bathed in a solution of antibiotic and he swallows the fluid.

Information on whether intrapartum ampicillin is preferable to penicillin for GBS colonized women is lacking. This study compared the efficacy of ampicillin and penicillin prevention, and found that ampicillin and penicillin are similarly effective in prevention for neonatal GBS-related outcomes (including GBS colonization, GBS infection, EOGBS infection, and mortality from EOGBS infection), indicating that ampicillin may be an acceptable alternative to penicillin for the prevention of neonatal GBS-related diseases. As penicillin had a narrower spectrum of antimicrobial activity and GBS continued to be susceptible to it, penicillin was the chief choice for IAP, with ampicillin as an acceptable alternative for the prevention of maternal infections. Notably, the ORACLE trial revealed that the prescription of amoxicillin–clavulanate or erythromycin for preterm labor (SPL) women was associated with an increased risk of cerebral palsy among their children at 7 years of age, supporting the opinion that antibiotics are not advisable in SPL without clinical signs of infection (Kenyon et al., 2008). Therefore, future studies must direct more attention to exploring the long-term effect of intrapartum antibiotics for neonatal adverse outcomes.

However, the increased use of intrapartum antibiotics to prevent GBS diseases has raised public health concerns over the emergence of antibiotic resistance among GBS strains. Several studies have reported that erythromycin and clindamycin resistance were the most common phenotypes (Jannati et al., 2012; Bolukaoto et al., 2015; Goudarzi et al., 2015; Kuang et al., 2015; Malek-Jafarian et al., 2015; Suhaimi et al., 2016). Since the first CDC guideline had been published in 1996, the frequency of erythromycin-resistant strains increased after a year (Lin et al., 2000). Although previous studies had not reported resistance to penicillin or ampicillin which was the first-line beta-lactam antibiotics, GBS with reduced penicillin susceptibility had been reported (Kimura et al., 2008; Jannati et al., 2012; Seki et al., 2015; Suhaimi et al., 2016). On the other hand, the increasing resistance to the macrolides and clindamycin which used as alternative drugs for penicillin allergic patients should be paid attention, and guideline for these patients need to be further study (Bolukaoto et al., 2015).

There are several potential limitations to this meta-analysis. First, we did not attempt to search for unpublished studies, believing that the rigor of the peer-review process guarantees the quality of a published trial, which could bring the publication bias. However, because not all trials are registered in public databases, a reliable estimate of the publication bias is not yet possible. Second, although statistical homogeneity did not exist for all outcomes, the question of clinical homogeneity is difficult to answer. There were differences in study design, population source, gestational ages, maternal conditions, study quality, and therapeutic regimens. However, the likelihood should be small, because no significant heterogeneity was observed in the subgroup analysis for gestational ages, maternal conditions, and study quality. Third, only 2 (15.4%, 2/13) RCTs are considered high-quality studies since it was difficult to conduct a double blind RCT study in this field. Finally, pooling of the English and Chinese literature together to extract the relevant studies may bring language selection bias and increase the heterogeneity of study participants. However, it brings more eligible studies and thus more information for evaluating the efficacy of IAP for neonatal GBS diseases, and there was no evidence of heterogeneity among studies on neonatal outcomes.

In conclusion, this meta-analysis shows that antibiotic prophylaxis is effective in interrupting vertical transmission of GBS and in reducing the incidence of GBS infections (including EOGBS infection). However, the potential preventive effectiveness of antibiotic prophylaxis to reduce neonatal LOGBS infection and mortality from GBS infection is inconclusive, given lack of evidence from well designed and conducted trials.

## Author contributions

SL and JH contributed equally to this work; conception and design of the study, XY, ZY, and SL; collection of data, SL and JH; analysis and interpretation of data, XY, SL, JH, ZC, and DG; drafting of the manuscript, XY, SL, and JH; all co-authors participated in the writing of the manuscript and approved the version submitted for publication.

## Funding

This work was supported by the Science and Technology Planning Project of Guangdong province (No. 2014A020212306).

### Conflict of interest statement

The authors declare that the research was conducted in the absence of any commercial or financial relationships that could be construed as a potential conflict of interest.
