# Supplementary material for: Antibiotic Prevention for Maternal Group B Streptococcal Colonization on Neonatal GBS-Related Adverse Outcomes: A Meta-Analysis
Source: Front Microbiol. 2017 Mar 17;8:374. doi: 10.3389/fmicb.2017.00374 (PMC5355432; doi:10.3389/fmicb.2017.00374)
Supplement: Supplementary file 1 [file Table1.doc]

Table S1. Characteristics of study participants

| Author, year | Characteristics of study participants |
| --- | --- |
| Matorras et al., 1991 | Screened at 17-42 weeks’ gestation with vaginal, rectal or both specimens positive for GBS, and included a few women with infectious symptoms. |
| Gervasio et al., 2001 | Screened at 19-43 weeks’ gestation with vaginal, rectal or both specimens positive for GBS, and included a few women with infectious symptoms. |
| EL Helali et al., 2009 | Screened at 35-37 weeks’ gestation with vaginal specimens positive for GBS. |
| Li and Meng, 2010 | Screened at 35-37 weeks’ gestation with vaginal, rectal or both specimens positive for GBS. |
| Yow et al., 1979 | Screened at the onset of labor with vaginal, rectal or both specimens positive for GBS. |
| Easmon et al., 1983 | Screened at 36 weeks’ gestation with vaginal, rectal or both specimens positive for GBS. |
| Lim et al., 1985 | Screened at 36 weeks’ gestation with vaginal cultures positive for GBS. |
| Boyer and Gotoff, 1986 | Screened among women who made prenatal visits with vaginal, rectal or both specimens positive for GBS. |
| Tuppurainen and Hallman, 1988 | Screened among women admitted to the clinic in labor or for induction of labor with vaginal specimens positive for GBS. |
| Shen et al., 2012 | Screened at 34-37 weeks’ with vaginal specimens positive for GBS. |
| Ma et al., 2014 | Screened at 34-37 weeks’ gestation with vaginal, rectal or both specimens positive for GBS, and included a few women with infectious symptoms. |
| Bai, 2014 | Screened at 35-37 weeks’ gestation with vaginal, rectal or both specimens positive for GBS, and included a few women with infectious symptoms. |
| Zhang et al., 2015 | Screened at 35-37 weeks’ gestation with vaginal, rectal or both specimens positive for GBS, and included a few women with infectious symptoms. |
| Yang, 2015 | Screened at 35-37 weeks’ gestation with vaginal, rectal or both specimens positive for GBS, and included a few women with infectious symptoms. |

GBS, group B *Streptococcus*.
